# Enhanced saccharification yields from rice straw by senescence-induced expression of a cytokinin biosynthesis gene in intragenic rice plants

**DOI:** 10.5511/plantbiotechnology.25.1209b

**Published:** 2026-03-25

**Authors:** Kano Miura, Hotaka Nishimura, Yukihiro Ito

**Affiliations:** 1 Graduate School of Agricultural Science, Tohoku University, 468-1 Aramaki Aza Aoba, Aoba-ku, Sendai, Miyagi 980-8572, Japan

**Keywords:** cytokinin, intragenesis, rice, saccharification, senescence

## Abstract

Controlling the digestibility of cellulosic biomass is important for its efficient use. We generated intragenic rice plants showing enhanced saccharification yield of rice straw. The rice cytokinin biosynthesis gene, *LONELY GUY*, under the control of the rice senescence-inducible *STAY GREEN* promoter, was introduced into the rice genome via particle bombardment. The rice-derived herbicide resistance gene *ALS(G95A)* was used as a selection marker gene. Regenerated intragenic rice plants with no foreign sequences showed enhanced saccharification yields from the leaves at harvest, whereas no significant differences were observed at the heading stage. Because the saccharification yields of rice straw are reduced after senescence, which is suppressed by cytokinin, we propose that the enhanced saccharification yields of intragenic rice plants are caused by the delay in senescence of the rice leaves due to the expression of the introduced cytokinin biosynthesis gene upon senescence.

Plant cell walls are the most abundant type of biomass worldwide, and their efficient use is expected to help overcome global warming and establish a sustainable society. Plant cell walls offer an alternative to fossil fuel resources and can be utilized as renewable materials for producing chemicals and energy when degraded into fermentable saccharides (Yoshida et al. 2021). However, the physical strength and chemical stability of plant cell walls prevent cost-effective conversion into saccharides. Several studies have been conducted to enhance saccharification yields from plant cell walls, and engineering plants that can be easily degraded into saccharides is necessary. Overexpression or silencing of a cell wall-associated gene in plants showed enhanced saccharification yields (Ahlawat et al. 2024; Cai et al. 2016; Fan et al. 2017; Furukawa et al. 2013; Furukawa et al. 2014; Gallinari et al. 2024; Iiyoshi et al. 2017; Llerena et al. 2024; Mira et al. 2024; Nigorikawa et al. 2012; Pawar et al. 2016; Sahoo et al. 2013). Mutations in genes associated with the structure and components of the plant cell wall also result in an enhanced saccharification yield (Dang et al. 2023; Laksana et al. 2024; Li et al. 2017; Wang et al. 2024; Wu et al. 2021; Ye et al. 2021). In addition, our previous study on rice showed that leaves and stems had higher saccharification yields at heading than at harvest (Abe et al. 2016). This indicates that the saccharification yield from rice straw decreases with senescence, and suppression of senescence may maintain the saccharification yield at the heading level.

Cytokinins are phytohormones that suppress senescence (Zwack and Rashotte 2013). The expression of a cytokinin biosynthesis gene under the control of a senescence-inducible promoter suppresses leaf senescence without causing morphological alterations (Gan and Amasino 1995). Constitutive expression delays senescence (Kuroha et al. 2009). We hypothesized that the senescence of rice straw would be delayed if a cytokinin biosynthesis gene was expressed upon senescence in rice, and that saccharification yields from the rice straw would be maintained after heading, even at harvest. Therefore, we examined this possibility using an intragenic approach. Intragenesis is a tool aimed at modifying a host organism using only its own sequences or those of closely related species capable of sexual hybridization. Through a combination of a regulatory sequence and a protein-encoding sequence from different genes, no foreign sequence is introduced into the host organism (Espinoza et al. 2013). Thus, the gene pool exploited using intragenesis is identical to that available for conventional breeding, and several surveys have shown higher public acceptance of intragenic than transgenic crops (Holme et al. 2013).

We used the rice cultivar Taichung 65 in all experiments, and amplified three rice genomic sequences using PCR. The first was the *STAY GREEN* (*SGR*) promoter, which includes a 3.1 kb upstream sequence from the translation initiation methionine codon (Sato et al. 2007). The *SGR* promoter directs senescence-inducible expression in rice leaves (Furukawa et al. 2014). The second was *LONELY GUY* (*LOG*), containing a 5.0 kb sequence from the translation initiation methionine codon to 1.3 kb downstream of the termination codon. *LOG* encodes a cytokinin-biosynthesis enzyme (Kurakawa et al. 2007). The two sequences were amplified using genomic DNA as a template. The third was the mutated acetolactate synthase gene *ALS(G95A)*, which confers resistance to bispyribac-sodium (Okuzaki et al. 2007). *ALS(G95A)* was amplified using the binary vector pSTARA380RALS(G95A) as the template, which contained *ALS(G95A)* as the selection marker. The primer sequences are listed in Supplementary Table S1. The three PCR fragments were cloned into the pBluescript SK- cloning vector using In-Fusion (Takara Bio) (Figure 1, Supplementary Figure S1). The plasmid containing SGRpro-LOG-ALS(G95A) was digested with the restriction enzymes *Nru*I and *Kpn*I, electrophoresed on an agarose gel, eluted from the gel, and introduced into the rice genome through particle bombardment of seed-derived calli using a Biolistic PDS-1000/He Particle Delivery System (Bio-Rad). Because the *Nru*I site and five nucleotides, except for the last nucleotide of the *Kpn*I site, are from the rice genome, SGRpro-LOG-ALS(G95A) digested with *Nru*I and *Kpn*I essentially consists of rice genome sequences. We obtained six regenerated plants that showed resistance to 25 nM bispyribac-sodium through the particle bombardment of rice calli with SGRpro-LOG-ALS(G95A). PCR analysis with two combinations of primers that specifically amplified SGRpro-LOG-ALS(G95A), but not an endogenous sequence, confirmed the presence of SGRpro-LOG-ALS(G95A) in the regenerated plants (Figure 2A, B, Supplementary Table S1). In addition, primer combinations that amplified the borders between SGRpro-LOG-ALS(G95) and the vector showed no bands from the regenerated plants (Figure 2A, C, Supplementary Table S1). These PCR analyses confirmed that the regenerated plants were intragenic.

**Figure 1:**
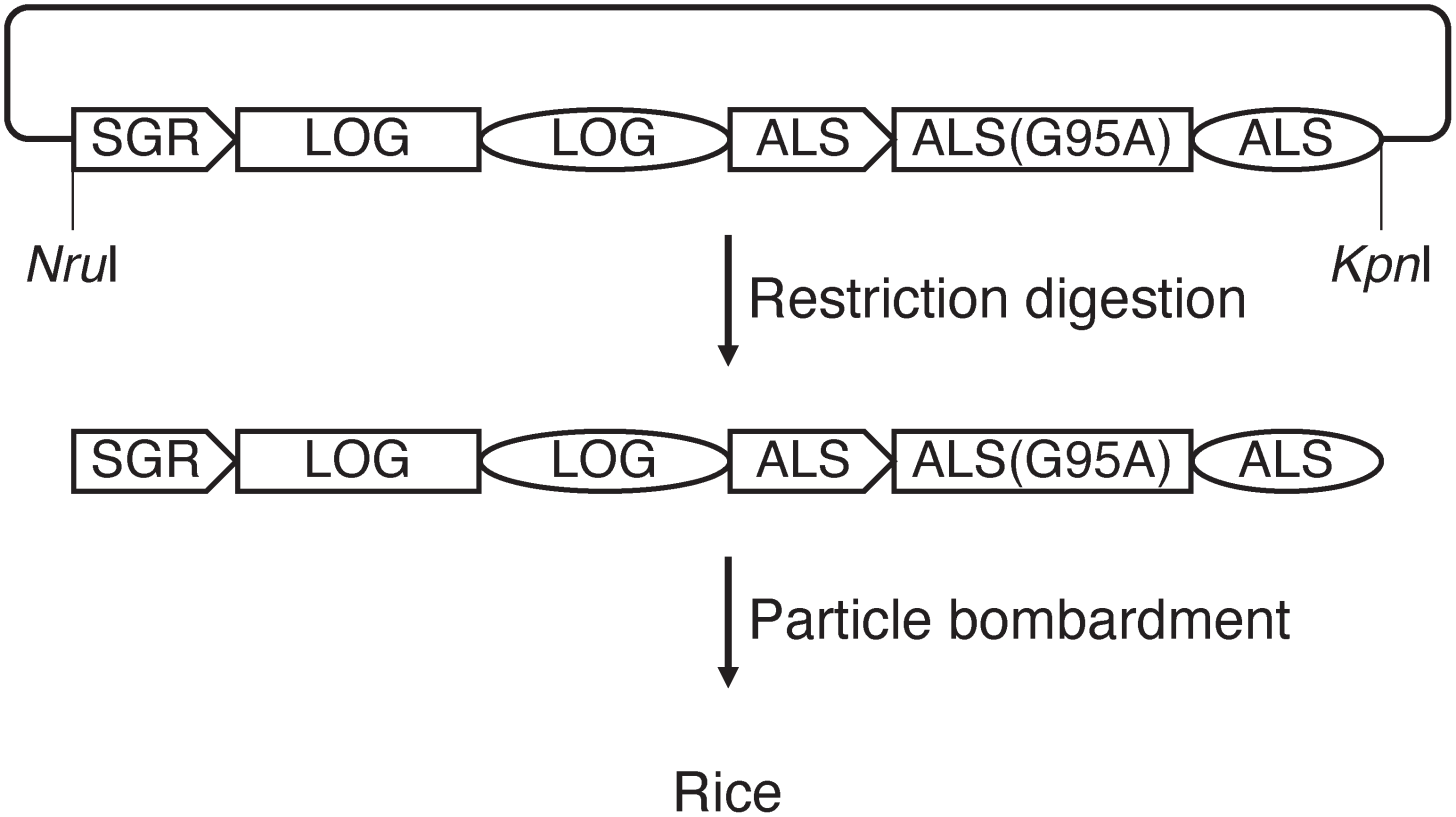
Figure 1. Strategy for generating intragenic rice plants. A plasmid containing SGR-LOG-ALS(G95A) was digested with restriction enzymes *Nru*I and *Kpn*I and electrophoresed on an agarose gel. The SGR-LOG-ALS(G95A) fragment was eluted from the gel and introduced into the rice calli through particle bombardment. SGR, SGR promoter; LOG, LOG coding sequence (square) and terminator (circle); ALS, promoter (arrow) and terminator (circle) of the acetolactate synthase gene; ALS(G95A), coding sequence of the acetolactate synthase gene with a mutation that replaces glycine at position 95 with alanine.

**Figure 2:**
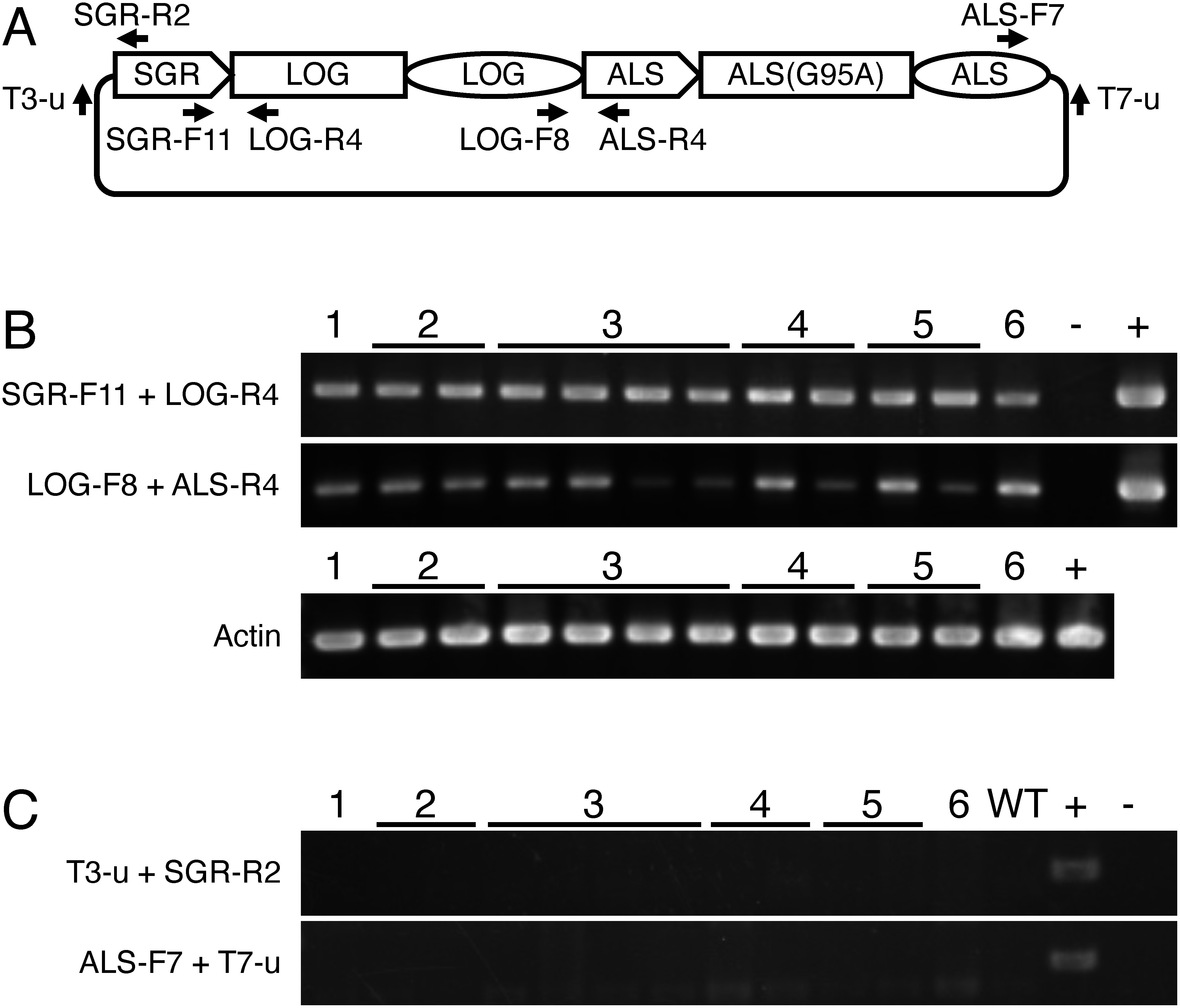
Figure 2. Genomic PCR analysis of the regenerated plants. A. The position and direction of the primers. B. Genomic PCR results confirming the presence of SGRpro-LOG-ALS(G95A) in the regenerated plants. C. Genomic PCR analysis confirming the absence of the vector sequence from the regenerated plants. DNA isolated from 1–4 leaf blades of each regenerated plant was used as a template. WT, wild type; +, a corresponding plasmid as a positive control; −, no template DNA. SGR, SGR promoter; LOG, LOG coding sequence (square) and terminator (circle); ALS, promoter (arrow) and terminator (circle) of the acetolactate synthase gene; ALS(G95A), coding sequence of the acetolactate synthase gene with a mutation that replaces glycine at the position 95 with alanine. Primer sequences are provided in Supplementary Table S1.

We examined the saccharification yields from rice straw at heading and harvest (40–50 DAH). Plants were grown in a phytotron under 10 h of light at 28°C and 14 h of darkness at 23°C. Saccharification was performed as previously described (Abe et al. 2016). Briefly, rice straws were divided into three parts (i.e., leaf blade, leaf sheath, and stem), dried at 105°C for 5 h, and ground into powder. Dried and ground samples were fractionated using mesh to collect particles with diameters less than 77 µm. The powder (15 mg) was incubated at 50°C for 48 h in a 1 ml reaction mixture containing 100 mM sodium citrate (pH 4.8) and commercially available cellulases (0.5 µl of Cellulase from *Trichoderma reesei* ATCC 26921 and 1.5 µl of Glucosidase from *Aspergillus niger*; both from Sigma-Aldrich), and the reducing sugars in the reaction mixture were measured using the DNS method. The saccharification yields were calculated by subtracting the values of the reactions without enzymes from those with enzymes. The results are shown as relative values, with the wild-type value set to 1.

No difference in saccharification yield was observed between the intragenic and control wild-type plants at heading (Figure 3). The leaf sheaths of three intragenic plants (#3, #5, and #6) showed significantly higher saccharification yields than those of the control plants at harvest (Figure 3). Although not statistically significant, two intragenic plants (#2 and #4) showed higher saccharification yields than the wild type. One plant (#5) showed significantly higher saccharification yields in the leaf blades, and four intragenic plants (#2, #3, #4, and #6) also showed higher saccharification yields in the leaf blades, although the differences were not significant. Intragenic plant #1 showed no enhancement in saccharification yield. No enhanced saccharification yields were observed in the stems, because the expression level of *SGR* in stems is not as high as that in leaves (RAP-DB: https://rapdb.dna.affrc.go.jp/index.html (Accessed Jan 29, 2026)). Thus, the activity of the *SGR* promoter in the stem is low. These results indicated that introducing SGRpro-LOG-ALS(G95A) enhanced saccharification yields from leaves at harvest.

**Figure 3:**
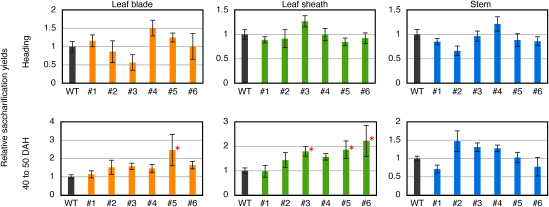
Figure 3. Enzymatic saccharification yields from rice straw. The blade and sheath of the flag leaf and the two leaves before the flag leaf and stem were dried, ground into powder, and subjected to enzymatic saccharification analysis using commercially available cellulases. The reducing sugars in the reaction mixture were measured by DNS. The relative saccharification yields of wild-type plants, set to 1, are shown as bar plots. Error bars indicate standard error. Asterisks indicate significant differences at the 5% level according to Dunnett’s test (*n*=4–15).

Next, we performed RT-PCR to examine the senescence-induced expression of the introduced gene *LOG*. RNA was isolated from the blade of the flag leaf at heading and 20 days after heading (DAH) because RNA isolation from old leaves at harvest (40–50 DAH) is difficult. PCR was carried out with 35 cycles at 94°C for 10 s, 55°C for 30 s and 72°C for 1 min using primers that anneal the 5′ untranslated region of *SGR* and the exon of *LOG* to detect only introduced *LOG* but not endogenous *LOG*. The results showed that *LOG* expression was detected at 20 DAH in four lines (#1, #3, #4 and #5) (Figure 4). Four lines (#1 to #4) showed *LOG* expression even at heading. This expression pattern does not seem to be consistent with the saccharification yields. However, there are two antagonistic explanations for the expression of *LOG*. One is that the expression of *LOG* induces delay of the senescence and thus the plant is not senescent, and the other is that the plant is senescent and thus *LOG* is expressed. The expression of *LOG* induces delay of senescence, which in turn reduces the expression of *LOG* driven by the senescence-inducible *SGR* promoter. This indicates that the expression level of *LOG* and senescence are on a delicate negative feedback balance, and the expression level of *LOG* does not simply reflect delay of senescence. In addition, the expression level of *LOG* seemed to be low, because 35 cycles of the reaction, which are generally assumed to be a large number to detect the expression of an introduced gene by RT-PCR, were necessary to detect the expression signal. An activity of *SAG12* promoter, which is another senescence-inducible promoter, was also very low, when a cytokinin biosynthesis gene was expressed by the *SAG 12* promoter in tobacco (Gan and Amasino 1995).

**Figure 4:**
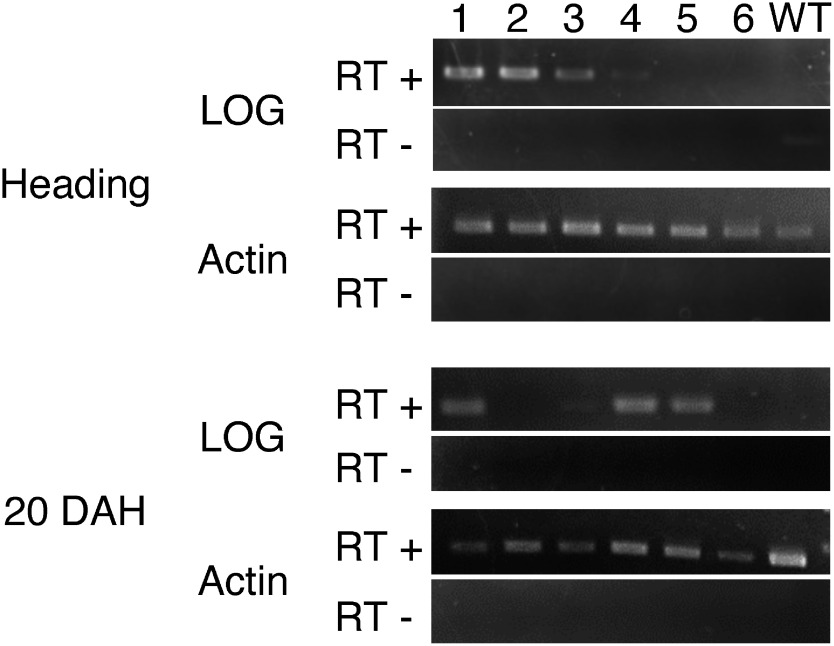
Figure 4. RT-PCR analysis of *LOG* in the regenerated plants. RNA isolated from the flag leaf blade was subjected to RT-PCR analysis using introduced *LOG*-specific or actin-specific primers. RT+ and RT− indicate that reverse transcriptase was added to or omitted from the reverse transcription mixture, respectively. 1–6 indicates independent intragenic plants. WT, wild type.

We generated intragenic rice plants with enhanced saccharification yields from rice straw at harvest. To the best of our knowledge, this is the first study to alter the recalcitrance of plant cell walls using an intragenic approach. Since the saccharification yield of rice straw is reduced after senescence and cytokinin suppresses senescence (Abe et al. 2016; Zwack and Rashotte 2013), the enhanced saccharification yields of the intragenic rice plants may be caused by the delay in senescence of the rice leaves due to the expression of the introduced cytokinin biosynthesis gene upon senescence. Further analyses of intragenic plants are necessary to uncover the mechanisms of reduction underlying the saccharification yields from rice straw, along with senescence, and the prevention of their reduction by the expression of cytokinin biosynthesis genes.

## Abbreviations

DAH: day after heading

## Description of supplementary files

Supplementary Table S1. Primers used in this study.

Supplementary Figure S1. Complete nucleotide sequence of SGRpro-LOG-ALS(G95A) with deduced amino acid sequences used for particle bombardment.
