# Supplementary material for: Enhanced saccharification yields from rice straw by senescence-induced expression of a cytokinin biosynthesis gene in intragenic rice plants
Source: Plant Biotechnol (Tokyo). 2026 Mar 25;43(1):145–9. doi: 10.5511/plantbiotechnology.25.1209b (PMC13170792; doi:10.5511/plantbiotechnology.25.1209b)
Supplement: Supplementary Data [file plantbiotechnology-43-1-25.1209b-s001.pdf]

Supplementary Table S1. Primers used in this study

| Primer name | Sequence | Purpose |
|---|---|---|
| ALS-F4 | CTTGCGCTGCGTTTGTGCGG | Amplification of ALS(G95A) |
| ALS-F7 | CTTCCTGCTGTCTAGGTTTG | Detection of the vector sequence |
| ALS-R2 | CTCACTATAGGGCGAATTGGGTACCCAATAAGATCGACCG | Amplification of ALS(G95A) |
| ALS-R4 | GATTGGATGAGGATGACAGG | Detection of the introduced sequence |
| LOG-F1 | AGAGATCCGAGGGAGCAGACATGGCAATGGAGGCTGCGGC | Amplification of LOG coding sequence and terminator |
| LOG-F8 | GTACTGAATTGGGAGAGTGT | Detection of the introduced sequence |
| LOG-R2 | CCGCACAAACGCAGCGCAAGGAGTTCGCTCTACGCATGCT | Amplification of LOG coding sequence and terminator |
| LOG-R4 | TAGCTGCCTAGGATACTAGG | Detection of the introduced sequence, RT-PCR |
| RAc-1 | AACTGGGATGATATGGAGAA | RT-PCR |
| RAc-2 | CCTCCAATCCAGACACTGTA | RT-PCR |
| RAc7-F1 | GCCATCCTGCGTCTGGACCTT | Amplification of actin |
| RAc7-R | AGGGCAGTGATCTCCTTGCTCAT | Amplification of actin |
| SGR-F7 | CTAGAACTAGTGGATCCCCCTCGCGAGAGAGGGCAAACCG | Amplification of SGR promoter |
| SGR-F11 | TACTGCTGGCTAGCTAGGAC | Detection of the introduced sequence, RT-PCR |
| SGR-R1 | GTCTGCTCCCTCGGATCTCT | Amplification of SGR promoter |
| SGR-R2 | GATTTCTTGGGTTGAGATGC | Detection of the vector sequence |
| T3-u | TTGTGTGGAATTGTGAGCGG | Detection of the vector sequence |
| T7-u | TAAGTTGGGTAACGCCAGGG | Detection of the vector sequence |

TCGCGAGAGAGGGCAAACCGTGCCTTCTAAGCATCACGGAAATGAAGCCCACACGCCGGTGCATCCACGTCGCCTAGCTAAATTTGGCCC 90

(*Nru*I) *SGR* promoter

GGTGTCCTCTCCAGTTTCCTGACGACGACTGCATCCTGGAACTGGAAGTAGTAGTAGTAAATAAAATACTCAAAATTCACCGGAATTCAC 180

CTCCGGATTACTACTAAAATGCATCTCAACCCAAGAAATCATCTCTATGTATTAAAAAGGAAAAAAAATACGAATTACCCTTTTAAACTA 270

TAGCGGTCGATCGAATTATCCCCCTAAACTCGAAAACCAGACATCGTTCCCCCTAAACTATGTATACCGGACGAATAACCCCCCCTGAGC 360

ACTGTTTTGGATGGTTTTTGACTTGTGTCTGCATACGTGGCCGCCAACGTGACATCCAGTCAGCAAAATAAAAATAAAAAAAATCTTAGG 450

ACCCATATGTCATCTCTTTCCTCCACCAATCCCTCTCAAATCCCATCCCCCTGGCCCCTGCCTCCCTGCTTGCTCTCTCTCTCTGCGTCT 540

ATGGCTGGAGGAGGAGACGGTGGCCGACGGCGGAGACGGATCGACGCGCAGACATCGGTGCACAAACCATCACCGAAGGTGGCTGCCGTG 630

CTTGTCCACCACCCCCATCGGCGACGTTGGCGAGCCTCCGCGGGAGCATGAGGCAGAGATGGGGGACAAGCACGATGTCCGGTGGAAGCA 720

AAGGTGGGGCGCGTCGTACGGTCACCCGAGCTCGATGGTAATGGGGGACTCGCGCCTTGCCAGCCGGCCACCACCACAGCAAGGGTAGAG 810

AGGAGAGAGATGGCGCGTCGCCGTGCGCCGGCCGGCTGCCGCCGCTCGCTGCAGCAGGTGAGGGAGGAGGAGGAAGAGAGGGGATGGGGA 900

TTGAGAGAGTAGAGAGAGAGATTGAGACGTTAGAGGTGAAAGAGAGAGCGGAGAGTCTTACAGGTGGGGCCCACACTGTTTTTTAAAAAAAA 990

AATGCTGATCGGACTGCCACGTAGTACTGAAACCACCCCGGATTGAGCCGGGGGTTATTTACCCGGTTTGTATAGTTAAGGGTGTAAAAT 1080

ATCTGATATTCGAGTTTAGGGGGTAATTCGATCGTCCATAATAGTTTAGGGGTAATTCGTAACCGTGGATTATTTACCGGACGGAATTCA 1170

CCTCTAGATTATTTACGGCCGGGCTGCAAATTCTCGCTCGGAGTTGCATGAACGTCTCCGCCTCTGACGAATCTGCACTTTCTGCAGCCT 1260

TCCGCCGGCAGCTAGCCCGCCTGGCGTATCGGAGCAGAATTTTTACAGATTTTTTGAGACATTGTGGCGTTTTGAAACAGTTATAACTGC 1350

AGTATATGTGATTCACGTTGGGCCTCAAGATTTTCAATTTTCCTTTTTAATAAGATTGGGTCCCCAGATTGAAGGTATATATGATCATTT 1440

CATATGGGGGTTCTTAAGCTGACCCATCGATATACGATTTTGTCTAGCTTATGTAGATTCTTAAAAGCTCGTTACCAGCTAGCTGTTTCT 1530

CATAGCGTTAACCTTCACAAACATGGCATACATATCGAAAACAGAAGCAAAGAAACGGATCGATGAAGCTTTGGCGCTCTCGTTCATCCT 1620

GTTTGGTCACGTGGGCTGTGGGAACGGGTGAGGCTAGAACATGATAACCACGAGCCAAGTCTCCCATTTTCGTCTTCCAACTATATGTCG 1710

CATGGCCGTATTTGCCAAACGTGACGAATGCTCCTTCTTCAGTTAGACCGAGAAAAATTAAGCTAGATTCCATAACTAGCATCGATCGCT 1800

TATGTGAGTGTGTCAGCTGCCTTGCACGAGCTGATCAGCTCGCACGCATCGCGGAGGATTCAGTCAAAAATATGATCACTCGCGCATATA 1890

TACATGCATTTCAGAAAGCACGGATGCGCCTAGCTCGCAAGCAACAGCGCCGTGCAGTAGCCTTACACCATGTAATTACACTTGGAATCT 1980

AGTTCTACCATGTAATTACACTTGGAATCTAGTTCTATAAAAAAAAGAAGTTGGAATCGTGTTTGGAACGATCACTCTGAAGTTTGATCC 2070

ACTCAGATCCCCCGGAAAAAAAAATATCTCCATCATTTGCTTTGTCAACGAGTTACTTGAAATGTTGAGTACACTGCGCGGTGCAACGTA 2160

CGTAACGCAAAATTACAATCCCAATTAAGCAGACGCAGTCCCCATATATTGCATGGTCAAAACCAAATGAAGTCCAAGTCTCCACTAGAT 2250

TAATTCGGGCCAATATTAAGTATCCTGCTGTATGTCTTTCGGTAGAAACTCTGCAAATTACGATCAATATTTAGCCCGTTTCAAGAGGAA 2340

AATAGTTACAGATAAGTAGGTGTGTGGTACGTATACGTATATATGTGTTTAGTTCACATCGAAATTGGAAGTTTGGTTAAAATTGAAACG 2430

ACGTGACAAAAAAAGTTAAAAGTTTATATACGTAGGAAAGTTTTGATATGATGAAAAAGTTGAAAGTTTGAAGAAAAAATATTTGGAACT 2520

AACAAGGCCGTAACCGAATGTCAAATTTGCATCAAACCACGCTCCTGTAGCAGAGACGCCACGTGCAGCACGGCCAAGCGTTCCATTCCT 2610

CCCGTTTCCACCCACGCGCGAACGCCACAATTTCCCCCGTTTTCCTGGGCGTTTTTCCACCACCACCACCACCGCCGCGAGACGACACGA 2700

GAGGAGGGCGCGGCGACGACGCGAGCTTCCCCGCGCGCGCACACAACCGCGACGTTTCCCGACCCCTCACTCCACCCCCCACCGCACCAC 2790

GCGTGCACACCAACACTAATACAACACTCTTCTTCTCACTTAACCCGCTCTCTCTTATAATCACCAACCTGCAGCGAGCTGAACTGACTG 2880

AGAAGCCAATCCGCGAAGCTACCAAACCGAGCCAAAATTTTGATCGATCGATCGGCCGGCCGGTGTCTTTGTTGAGGCGTGCAAGGCTCG 2970

TGCTTTCGCCGAGAGGGATTAATTAGCCGCGCCTCCAGCATCTCCGGCCTCCATAGAGATCCCAGCTTATAATTAAAGCTGTCAAGAGTC 3060

GTTTTGGTTGTTAGGCTTAAAGTTAACTACTGCTGGCTAGCTAGGACTAAGAGATCCGAGGGAGCAGACATGGCAATGGAGGCTGCGGCG 3150
(LOG) M A M E A A A

GAGAGGAGCGCCGGAGCAGGGGCGGCGGCGACGGCGGCGCCGGAGAGCGGTGGCGGTGGTGCAGGGGAGAGGCGGTCGCGGTTCAGGCGG 3240
E R S A G A G A A A T A A P E S G G G G A G E R R S R F R R

ATCTGCGTGTACTGCGGCAGCGCCAAGGGGAGGAAGGCCAGCTACCAGGACGCCGCCGTCGAGCTCGGCAAGGAACTGgtcaggattctt 3330
I C V Y C G S A K G R K A S Y Q D A A V E L G K E L

cttcttcttcttcttcttcttcttcttcttcttcttctttttttgatttgattgattgtcctttctccctccctccctcccaaatccg 3420

tcataaaaatccaatcttttcattttctacttttctttctttcttccccctccgggcttcgccggccggctcgccggcggcggcggc 3510

gaccgactctccggctcgccgtggccgtttctctcttggtgcgcgtccctagtatcctaggcagctatagtagtaggttttctagatgag 3600

caaacaaaaccaatcatgattgctggcgagcgcggtgacctcagttgcgtcccgaacggaacaacaatcttacaatttttgcatgcatca 3690

aaacgccaccaccatctcctccctctctcccccaccttttggatttttcttgtcgactcaagagagtagagagaggttgattggtttctt 3780

gttttttacatttccccttttgtttttggttcgtgttggtgcagGTCGAGAGGGGCATAGACCTGGTCTACGGCGGTGGCTCCATCGGC 3870

V E R G I D L V Y G G G S I G

CTCATGGGCCTCGTCTCCCACGCTGTTCACGACGGTGGTCGCCATGTCATTGGgtgagtgatccatttgtccatctctctctctctctat 3960

L M G L V S H A V H D G G R H V I G

ctttctctggttgcttgccaggtgagatgtgtgagaatgcatgcgtcgctgggatgataaaaacggcagcttttgatcgccagattgggg 4050

gtaggaggagtagtaggtgctgagtccctgcccattttcgtctctctttcttgggtctcagtgtgggggggggggggcattttgaatgc 4140

atctggggccttttcgcctccgtttccgaaaggctactcgcctgcatgcttgaattgttgtttgtgcagcagcacactccaacttcactt 4230

ggcctgctcctgcatagctcagctcctgcaatctgctgctgctgctgctgctgctagcttgctagtactagtgctcttctcttctttgct 4320

ctcattattgtgcagtgtttatttggtccagctccctctctctctctatctatctgtattgctagtgtggtgtagctgtgctgcctgtgc 4410

aatagcctgggccttggctttgccagtctctttaccaagggagagaaagcaagcctgcttgttcttactacacctgtttgaccgcttttg 4500

tgctaaaaactgatgtgcttttctgtttcctctctctttgtgttcttggcagGGTCATCCCGAAATCCTTGATGCCCAGAGAGgtgagct 4590

V I P K S L M P R E

gtctactcctggaagctagtaggagtagtactactgctattacacaagctcgatcgatagagatagatataatcttgattgctgtttta 4680

atgtggccgtgctgctccattttgttcggggcgaaattgatgaatgctttcaattcctctccctgctggcttctgctgctgctgctgctg 4770

ctgtgcgcagctgaaaaataggagttgcaataaagaaccaagttggcatgctgctagtgcttattggggggacatgatgcatttatgctc 4860

atcacatgaatctaatgcgcctatccaacagcattgcgtgctgctcttaaatggattttgagctatagtagctgtctaggatgtgccatc 4950

gcatttgccttgccttcggataatttactggggcttgcacgtagtagtaagtgcagtaactgggattatttactcttcttgtacgtatct 5040

ctacttgtactagtactcattcggtagtacaatgtcttaaaaatgggactttcttgattgtcgtggtctttgaccactgcattacgtacg 5130

tagaacttgtatttcctgtgtattctaatgtcgatgatgatctcacgctgaacaactctgtgtgccgtagtttgcatccgaatgttgtca 5220

gccatgtatttctatggacttttgcattgctagtttcactaaaaaaggtttttaaatatttttctactaggatgatgagcttattaacat 5310

gattctgaaagagaaacaagtgtgatgtgtgccggatcctcaatatatactggtttttcgtttgcagGTCACTGGTGAGCCTGTTGGTGA 5400

V T G E P V G E

AGTTAGAGCGGTCTCTGGCATGCACGAGAGGAAGGCTGAAATGGCCCGGTTTGCTGATGCGTTCATTGCACTGCCTGgtatgataattta 5490

V R A V S G M H E R K A E M A R F A D A F I A L P G

gttgctactacgacgtcgtttgcagtgaatttttttttgatgctgcatcctaaggatgcaaaatttgacactcttaatttgcgcgacagGC 5580

GGCTACGGGACTCTTGAGGAGTTGCTTGAGGTCATCACCTGGGCCCAACTAGGAATCCACAAGAAGCCGgtaagtaaccaactggttctg 5670

G Y G T L E E L L E V I T W A Q L G I H K K P

attcctctgtatatcttgtactagtaagattcacgaagaaaacgaatggcattattgcgcgataatttggaggccatgtggttgtgcgtg 5760

catgatcccgaattcctttcttgtctctggttgcaattgaggcaaattaataatcgcaaagtcgttggatccattgagatcttgtgctaa 5850

catcttgcccttgattatggcttcgtggtgctcccatgtggttggctgaaggttttgccatctagaaaccgaattattgaccaatagttt 5940

aagccctctatgatctgcagatcttacagtacattaggcgatagcttggggatcacgagatctgcaccctctattgtttatgcttcctgt 6030

taatatgatatatgctttatacttttatgaatgttccaactcatctaatagaatgatgttcgcaggatcttgtacctgtcattttttta 6120

attcaaaaaggaacttatcgtttgcatctatgctaaattaatgacccaatggatctgagcagtgcattaaacctcattcatcatgcctag 6210

caatcagctttgctgatggcccccacaaaattcatatggtgtcgtgtaactgtattaactaggatagtgatccttcttggatccatgttt 6300

ttagaggaatgctcgtcttttaatcgcgcgggatttcccttattgctctcaaatgcacattctctactcctagctctaacagctcctcaa 6390

tatttaccagGTTGGCCTTCTGAATGTCGACGGGTTTTATGATCCTTTTCTATCCTTCATTGACATGGCTGTCAGCGAAGGATTCATAGC 6480

V G L L N V D G F Y D P F L S F I D M A V S E G F I A

GGAGGATGCGCGGCGCATTATCATCTCGGCTCCAACTGCCAGGGAGCTAGTTCTGAAGCTTGAGgtaaaaaaaactcacagcttgcactg 6570

E D A R R I I I S A P T A R E L V L K L E

ttccattttcgacacattgccctacatggcaatttccttctcataacaccaagtttgtatgtacccttttggaaatttcagGAGTATGTT 6660

E Y V

CCCGAGTACGAGGTCGGCTTGGTTTGGGACGATCAGATGCCGCACAGCTTCGCGCCTGACCTCGAGACCAGGATCACCTCATCCTGATCT 6750

P E Y E V G L V W D D Q M P H S F A P D L E T R I T S S *

CTCGGCTCTGGCCCTATTCTTACCCAAGCTTCTAACCGGCGGACGACGATACCTTCTTCTCGTGCCGAAGCCGAATATGATCCTGGAGGA 6840

AGTTTGTTTTTCTCAGCCAGGGGAAAGTAACATCCACTAACCGAAAAGATTTAGCCAAAGGCGGCCCCTAGCTCGGGTCTGCCGATTTGT 6930

TGTTTTGGCAAAATATGTCAATTGGGACAGTATTTAGCATTAGCAGTGCCTCCTCAATGCTGTTCTTTTGGCCCCCCTCCCTCCTCACTA 7020

GCTAGAAGAAGGAATATATGTATATGCAAGTAATATTAGTAGCATCTGTCTATCTTGATCTTCTCCATATGCCTGTGCAATCTGTGTCCC 7110

CTTGTTTGATCCGATTCTGCACTGAAATTGATCATGCACGTGTGGATGGATGGATTCGGATTCAGAACTGATTCCTGGAAATGATTTGAT 7200

GATTCGTCTCTAAAAGAAAAGAATCTCTGAAAGTCTGAACCATGTTTGACTAACTGAAGAAATGTGTCTCATCTATTGCTGATGGAAAAG 7290

GTGGTCCGGGTCCGGGCTTTCCGGCTGGCCGGCCGGCGTCGCCGTCGGCAGCGATGGGACGTGCCGTCGCCGGTTGTCGGCAAGGGACAG 7380

GGAGGCAGCCGGTGGGGCTCGCCGGGGCGACGAATTGGGTCACCGGATTTGGACGTGCCGTTGCTCCTGCCCTTTCGGTTGGTTAGGTTG 7470

GTTGGTTTGTTGGTTGGTTTGGTTCCTGCGTTTGATTCCAATTCCAAAGGGGGGAGGTTGACATTGGAATTCTGGTGTTCCCTGGCCTGA 7560

GGAAATGCAGGGCAAAGATGGTGATGACTTGTGATTTATATATAAAGAAAAAGGAGATGGTGATGACTTTGTGATAGGTTTTCACTTGTG 7650

GGACCTTTTATTTTTCTTCTTCTGAAAATCCAACTTTTTGGTCTTTGGTTGATGTTTGATTCTAGTACTGAATTGGGAGAGTGTTAGAGC 7740

TAACATATACTCCCTCCGTTTCAAAATGTTTGACACCGTTGACTTTTTAGCACATGTTTGACCGTTCGTCTTATTTAAAAACTTTTGTGA 7830

AATATATAAAATTATATGCATACATAAAATATATTTAACAATGAATCAAATGATAATCAAATGATAGTAAAAGAATTAATAATTACTTAA 7920

ATTTTTTGAATAAGACGAACGGTCAAACATGTGCTAAAAAGTCAACGTAGTCAAATATTTTGAAATGGAGGGAGTATAATATCTTAACTA 8010

TAGGGTACGGTTGGCTTTATTTTTTTTTCTCTCCTAAATTTAATATAAATATTTTGGAGCATGCGTAGAGCGAACTCCTTGCGCTGCGTT 8100

ALS promoter

TGTGCGGGTGCGGGTGCGGGTGCTAGACTGCTAGGTCTCGCGGTTGCATCCGCATCCGACTTTGAGATCGATTTTTTATCGGGTTCTGTA 8190

CCCTCCACCCGTTATTGGGACTGACCCACCTGTCATCCTCATCCAATCGACTGACACGCGGGCCCAGATCGACCCCGACGTGGCTGTGTG 8280

TCATCCTATCCCACCGACATATGGGGCCCACTGTGACGTGGCCCCACACGATCCCATCCGAGCCACACATCGCCTCACGCTGCGTCACCG 8370

CGCGCGGACAAAACACCCACACCCCCACACTCTCCACCCCTCTCTCCCTCTCGCCCAAACCCAGAAACCCTCGCCGCCGCCGCCGCCACC 8460

ACCCACCATGGCTACGACCGCCGCGGCCGCGGCCGCCGCCCTGTCCGCCGCCGCGACGGCCAAGACCGGCCGTAAGAACCACCAGCGACA 8550

M A T T A A A A A A A L S A A A T A K T G R K N H Q R H

ALS(G95A)

CCACGTCCTTCCCGCTCGAGGCCGGGTGGGGGCGGCGGCGGTCAGGTGCTCGGCGGTGTCCCCGGTCACCCCGCCGTCCCCGGCGCCGCC 8640

H V L P A R G R V G A A A V R C S A V S P V T P P S P A P P

GGCCACGCCGCTCCGGCCGTGGGGGCCGGCCGAGCCCCGCAAGGGCGCGGACATCCTCGTGGAGGCGCTGGAGCGGTGCGGCGTCAGCGA 8730

A T P L R P W G P A E P R K G A D I L V E A L E R C G V S D

CGTGTTCGCCTACCCGGGCGCAGCGTCCATGGAGATCCACCAGGCGCTGACGCGCTCCCCGGTCATCACCAACCACCTCTTCCGCCACGA 8820

V F A Y P G A A S M E I H Q A L T R S P V I T N H L F R H E

GCAGGGCGAGGCGTTCGCGGCGTCCGGGTACGCGCGCGCGTCCGGCCGCGTCGGGGTCTGCGTCGCCACCTCCGGCCCCGGGGCAACCAA 8910

Q G E A F A A S G Y A R A S G R V G V C V A T S G P G A T N

CCTCGTGTCCGCGCTCGCCGACGCGCTGCTCGACTCCGTCCCGATGGTCGCCATCACGGGCCAGGTCCCCCGCCGCATGATCGGCACCGA 9000

L V S A L A D A L L D S V P M V A I T G Q V P R R M I G T D

CGCCTTCCAGGAGACGCCCATAGTCGAGGTCACCCGCTCCATCACCAAGCACAATTACCTTGTCCTTGATGTGGAGGACATCCCCCGCGT 9090

A F Q E T P I V E V T R S I T K H N Y L V L D V E D I P R V

CATACAGGAAGCCTTCTTCCTCGCGTCCTCGGGCCGTCCTGGCCCGGTGCTGGTCGACATCCCCAAGGACATCCAGCAGCAGATGGCCGT 9180

I Q E A F F L A S S G R P G P V L V D I P K D I Q Q Q M A V

GCCGGTCTGGGACACCTCGATGAATCTACCAGGGTACATCGCACGCCTGCCCAAGCCACCCGCGACAGAATTGCTTGAGCAGGTCTTGCG 9270

P V W D T S M N L P G Y I A R L P K P P A T E L L E Q V L R

TCTGGTTGGCGAGTCACGGCGCCCGATTCTCTATGTCGGTGGTGGCTGCTCTGCATCTGGTGACGAATTGCGCTGGTTTGTTGAGCTGAC 9360

L V G E S R R P I L Y V G G G C S A S G D E L R W F V E L T

TGGTATCCCAGTTACAACCACTCTGATGGGCCTCGGCAATTTCCCCAGTGACGACCCGTTGTCCCTGCGCATGCTTGGGATGCATGGCAC 9450

G I P V T T T L M G L G N F P S D D P L S L R M L G M H G T

GGTGTACGCAAATTATGCCGTGGATAAGGCTGACCTGTTGCTTGCGTTTGGTGTGCGGTTTGATGATCGTGTGACAGGGAAAATTGAGGC 9540

V Y A N Y A V D K A D L L L A F G V R F D D R V T G K I E A

TTTTGCAAGCAGGGCCAAGATTGTGCACATTGACATTGATCCAGCAGAGATTGGAAAGAACAAGCAACCACATGTGTCAATTTGCGCAGA 9630

F A S R A K I V H I D I D P A E I G K N K Q P H V S I C A D

TGTTAAGCTAGCTTTACAGGGCTTGAATGCTCTGCTACAACAGAGCACAACAAAGACAAGTTCTGATTTTAGTGCATGGCACAATGAGTT 9720

V K L A L Q G L N A L L Q Q S T T K T S S D F S A W H N E L

GGACCAGCAGAAGAGGGAGTTTCCTCTGGGGTACAAAACTTTTGGTGAAGAGATCCCACCGCAATATGCCATTCAGGTGCTGGATGAGCT 9810

D Q Q K R E F P L G Y K T F G E E I P P Q Y A I Q V L D E L

GACGAAAGGTGAGGCAATCATCGCTACTGGTGTTGGGCAGCACCAGATGTGGGCGGCACAATATTACACCTACAAGCGGCCACGGCAGTG 9900

T K G E A I I A T G V G Q H Q M W A A Q Y Y T Y K R P R Q W

GCTGTCTTCGGCTGGTCTGGGCGCAATGGGATTTGGGCTGCCTGCTGCAGCTGGTGCTTCTGTGGCTAACCCAGGTGTCACAGTTGTTGA 9990

L S S A G L G A M G F G L P A A A G A S V A N P G V T V V D

TATTGATGGGGATGGTAGCTTCCTCATGAACATTCAGGAGCTGGCATTGATCCGCATTGAGAACCTCCCTGTGAAGGTGATGGTGTTGAA 10080

I D G D G S F L M N I Q E L A L I R I E N L P V K V M V L N

CAACCAACATTTGGGTATGGTGGTGCAATGGGAGGATAGGTTTTACAAGGCGAATAGGGCGCATACATACTTGGGCAACCCGGAATGTGA 10170

N Q H L G M V V Q W E D R F Y K A N R A H T Y L G N P E C E

GAGCGAGATATATCCAGATTTTGTGACTATTGCTAAGGGGTTCAATATTCCTGCAGTCCGTGTAACAAAGAAGAGTGAAGTCCGTGCCGC 10260

S E I Y P D F V T I A K G F N I P A V R V T K K S E V R A A

CATCAAGAAGATGCTCGAGACTCCAGGGCCATACTTGTTGGATATCATCGTCCCGCACCAGGAGCATGTGCTGCCTATGATCCCAAGTGG 10350

I K K M L E T P G P Y L L D I I V P H Q E H V L P M I P S G

GGGCGCATTCAAGGACATGATCCTGGATGGTGATGGCAGGACTGTGTATTAATCTATAATCTGTATGTTGGCAAAGCACCAGCCCGGCCT 10440

G A F K D M I L D G D G R T V Y *

ATGTTTGACCTGAATGACCCATAAAGAGTGGTATGCCTATGATGTTTGTATGTGCTCTATCAATAACTAAGGTGTCAACTATGAACCATA 10530

TGCTCTTCTGTTTTACTTGTTTGATGTGCTTGGCATGGTAATCCTAATTAGCTTCCTGCTGTCTAGGTTTGTAGTGTGTTGTTTTCTGTA 10620

GGCATATGCATCACAAGATATCATGTAAGTTTCTTGTCCTACATATCAATAATAAGAGAATAAAGTACTTCTATGCAATAGCTCTGAGTT 10710

AAGTGTTTCAACAATTTCTGAACTTCTGAACTTATGTTTGCTCAACTGTCATCACACGAAGTACTCTCCTTGTAACTACATTTTCCCCAA 10800

GACTTTAAATCCCCTCAGTTACAGCAAAAAATAAACTTTGCATCTACTGTTTTCCCTCTCTTCGGTCGATCTTATTGGGTAC(C) 10882

(*Kpn*I)

Supplementary Figure S1. Complete nucleotide sequence of SGRpro-LOG-ALS(G95A) with deduced amino acid sequences used for particle bombardment.

*SGR* and *ALS* promoters and their 5′ untranslated regions were underlined. Translation initiation by methionine in LOG and ALS is underlined. Restriction sites used for plasmid digestion and release from the vector sequences are underlined. The introns are indicated by lowercase letters. The last nucleotide, 'C' in parentheses, was added to create the *Kpn*I restriction site and removed using *Kpn*I digestion.
